# Major Chapple, M.P.'s, Reply to Mr. Morris

**Published:** 1918-10-05

**Authors:** W. A. Chapple

**Affiliations:** House of Commons


### The London Hospital's Nurses.

**Correspondence on all subjects is invited, but we cannot in any way be responsible for the opinions expressed by our correspondents, who must give their name and address as a guarantee of good faith, but not necessarily for publication. Correspondents are reminded that brevity of style and conciseness of statement greatly facilitate early insertion.]**

### Major Chapple, M.P.'s, Reply to Mr. Morris.

*To the Editor of* The Hospital.

Sir,—Mr. Morris admits my facts and calls me a liar. He says, "Yes, we pay a 'private staff' nurse in her third year after entering the hospital £35 a year (13s. 5d. per week), and it is true that while she is nursing a case in private she earns *for the hospital* £2 12s. 6d. a week." I note that Lord Knutsford is in Scotland. I hope you will send him a copy of The Hospital on his return.

He says further, "The profit [to the hospital] amounts to over £15 and under £20 for each nurse engaged, after deducting expenses." What an unblushing confession of the exploitation of women labour! According to Mr. Morris these nurses are as fully trained as the three-year certificated nurses of Guy's and Bart.'s and St. Thomas's and all the other great hospitals in Britain; but the fully-trained nurses of these hospitals are free to leave and earn the £2 12s. 6d. or more for themselves. London Hospital nurses, on the contrary, are under a bond to "farm" for the hospital for two years after that hospital claims they are fully trained and certificated!

This claim is monstrous, and every hospital in London and Britain knows it. The claim is made, not because it is true, but because the nurses' exploiters make money out of them.

During the third year, when all other great hospitals in Britain are training their nurses in the wards, the London Hospital, alone in this iniquity, are depriving their nurses of their hospital training and robbing them of 39s. per week of their earnings while they are out nursing.

I do not hesitate to say that there is not a docker on the wharf, a groom in a stable, a fireman on a railway, or a lumper in a shipyard, who would submit to such treatment. Nor is there, I am sure, an employer outside the London Hospital so cruel and callous, and so lost to every instinct of chivalry, as to impose this oppressive tyranny on young and defenceless women. Mr. Morris refers to the "House Committee of the London Hospital." Who are they? Let us have their names. Are they aware of the facts that Mr. Morris makes public—when Lord Knutsford is in Scotland?

Mr. Morris says "that the 'London' can train in two years, partly because of its size and wealth of material." This is an unwarranted belittling of all the other great hospitals of London, England, and Scotland, all of which give no certificate of training under three years, and no one knows it better than he. Of what use to a nurse are one thousand patients in a hospital, if she, in twenty-four hours, can only see, attend, or dress a dozen or so? Mr. Morris complains that what I say is untrue when I state that the two years' certificate of training "only serves to enable the London Hospital to advertise in the *Times*," etc. This is true. No nurse is allowed to go off with her two years' certificate. It requires completion. It is not fully completed till she has finished her two years' "service" in addition to her two years' training. She cannot, therefore, use her two years' training certificate. This double-barrelled certificate is signed up as to two years' training at the end of two years, and as to two years' "service" at the end of four years.

She gets no certificate at all if she leaves before the four years are up. She has broken her contract. Her two years' certificate can be of no use to her whatever, therefore, in her third year. If "she still remains at the hospital" she cannot use it, and if she leaves the hospital she cannot take it with her. But it "serves to enable the London Hospital to advertise in the *Times*, "Trained nurses can be had immediately." Why did not Mr. Morris, instead of saying that what I said was "untrue," admit this? Lord Knutsford is in Scotland.

Mr. Morris tells us further that one of the unparalleled benefits for which London Hospital nurses are willing to endure present exploitation is the promise of a pension after eighteen years' service in the hospital. If he replies before Lord Knutsford returns from Scotland, will he tell us how many nurses (if any) are enjoying this fruit of their patience and this recompense of their persecution?

He talks of the cost of training. But nurses work for their training, and all other great hospitals are content with this and do not manœuvre their nurses in order to secure their earnings as well as their work.

Mr. Morris says the London Hospital nurses "still regard their work as a mission, and not simply a trade." Quite so. They deem it a mission: the hospital makes it a trade. Fie!—Yours faithfully, W. A. Chapple.

House of Commons, September 29, 1918.

